# Using contact tracing from interlocking diaries to map mood contagion along network chains

**DOI:** 10.1038/s41598-022-07402-1

**Published:** 2022-03-01

**Authors:** Yang-chih Fu, Ta-Chien Chan, Yen-Hua Chu, Jing-Shiang Hwang

**Affiliations:** 1 Institute of Sociology, Academia Sinica, Taipei, Taiwan; 2 Research Center for Humanities and Social Sciences, Academia Sinica, Taipei, Taiwan; 3 Institute of Public Health, School of Medicine, National Yang Ming Chiao Tung University, Taipei, Taiwan; 4 Institute of Data Science and Engineering, National Yang Ming Chiao Tung University, Taipei, Taiwan; 5 Institute of Statistical Science, Academia Sinica, 128 Academia Rd. Sec. 2, Nankang 115, Taipei, Taiwan

**Keywords:** Epidemiology, Human behaviour

## Abstract

Both viruses and moods are transmitted through interpersonal contacts, but it has been extremely difficult to track each unique chain of contacts through which particular moods diffuse. By analyzing 56,060 contact records from 113 interlocking, yearlong diaries collected through a web-based platform in Taiwan, we traced mood states before and after each specific contact along a triplet of persons where B contacts C and subsequently contacts A. Multilevel analyses show that both positive and negative emotions are contagious, but the two paths diverge markedly in how the diffusion stops. Positive contact between C and B (which leads to improved mood for B) spreads to A through B’s contact with A, making A feel better afterward, regardless of whether B’s mood deteriorated between the two interactions. Negative contact between C and B (which leads to worsened mood for B) also spreads to A, making A feel worse after the contact with B. However, the spread of a negative mood discontinues if B’s mood improved between the two contacts. The different patterns of diffusion suggest that a negative mood is harder to disperse, probably because people generally make efforts to keep their negative emotions from spreading to others.

## Introduction

Mood contagion resembles the transmission of influenza (flu) in some ways. Just as an infected person can pass the flu virus to others through interpersonal contacts, the extent and speed of flu transmission can vary depending on the viral load^1^, immunity of the susceptible person^2^, and patterns of in-person contacts^3,4^. Likewise, the spread of personal moods can also differ in pace and efficiency by emotional valence^5^, the relations between the persons^5,6^, and the social context surrounding an interpersonal contact.

Because both viruses and moods are transmitted from person to person through specific contacts, it is essential to explore the patterns and paths of flu infection and mood contagion by not only identifying interpersonal connections but also tracking each unique contact through which the virus or specific mood transmits. Based on such a contact-based approach of social network studies, we aim to use contact tracing from interconnected contact diaries to show how moods spread, or what we call mood contagion, along the chains of personal networks.

As the risk of getting the flu increases if one comes into contact with an infected person, we propose that the chances of mood change increase when one comes into contact with another person who changed moods during a previous contact with a third person. By spreading from person to person, mood changes can trigger a series of contact effects among network members. Similar network effects have been tested and verified in longitudinal research, including panel surveys among adolescents^7,8^ and archival studies of adults^9^. Thanks to the longitudinal design, these panel studies have shown that friends’ and relatives’ personal moods can concur over time. Because these studies included waves of interviews that spanned several years, however, the periods between surveys are often too far apart to reveal how personal moods might shift from moment to moment. As a result, it has been infeasible to identify precisely how moods spread^5,10^ without being able to consider fined-grained temporal changes.

To further investigate the network effects on mood contagion that we believe are embedded at a deeper level than interpersonal ties, we track how personal moods change along a series of contacts. Using “contact” as the unit to trace mood changes, our analysis also includes the information about ties and individuals embedded in the network data we collected by years-long contact diaries. In our diary study between 2017 and 2019, a total of 171 participants recorded their moods before and after 313,347 one-on-one contacts. Using data of 56,060 contacts from 113 “interlocking” contact diaries, we conduct multilevel analysis of sequential contacts and social connections among participants who were acquaintances. By incorporating the analysis of network structures into the step-by-step contact tracing of mood changes, the current study follows the contact-based network perspective to show how moods spread via certain contact patterns.

### Tracing mood contagion by contact patterns

Precise contact patterns help capture how viruses or moods flow along a series of interactions. Though it is clear that flu or other respiratory infections spread by close interpersonal contacts, the speed and scope of transmission may differ sharply depending on how the virus spreads and the underlying patterns of social interactions. An examination of such interaction patterns often involves more details about “social mixing,” or who contacts who and under what circumstances in terms of various network structures^11–13^. While highly mixed contact patterns tend to promote virus transmission in general, certain control strategies that reduce social mixing in the critical early stage of a pandemic (such as COVID-19) could substantially delay the epidemic’s peak^14^.

In addition, when individuals have more information about local transmission of diseases, they can alter virus transmission by changing their contact patterns or taking other precautionary health measures. Like the disease itself, such awareness is also likely to diffuse along network chains^15^, in a way similar to how critical information or job leads circulate from contact persons to help one secure a good job^16–19^. Unlike virus transmission, both disease awareness and moods can disperse through in-person contact or online communications^20–24^, thus making it more complex to track how they spread step by step.

As with the case of flu infection, mood contagion follows a chain of contacts with others. To track whether a certain mood state transmits from person to person, it is critical to examine the extent to which a person’s mood changes in the same direction after interacting with someone who has experienced either positive or negative mood changes from a previous contact with a third person. However, mood contagion may occur spontaneously or instantly during or after interpersonal contacts. It is thus particularly challenging to capture such spontaneous and instant contagion given the contingent nature of social interactions.

### Unique features of mood contagion

When people interact with others who have good or bad moods, their moods also tend to change. It remains unclear, however, whether such an association of mood states between a pair of actors is indeed due to the contagion of personal moods. Although previous research has examined how various moods could “transfer” between persons, or how one’s behavior could influence others’ behavior^21,22^, our study attempts to identify the process by which a person’s mood changes during or after interacting with another person whose mood also changed during or after a preceding contact with a third person. In order to track mood contagion more precisely, therefore, we need a more thorough understanding of how personal moods spread through a series of two or more consecutive social interactions among a triplet of persons.

In addition to its spontaneous effect, mood contagion is also unique in the chains of transmission. While a virus may transmit from person to person when people are in physical proximity^13,23^, personal moods can spread through various forms of communication without seeing or being with others in person. Besides interpersonal contacts, the conditions under which mood contagion occurs may be more diverse and can vary depending on social relationships^10,24^. In other words, although both flu infections and mood contagion follow through “contact chains,” the chains of mood contagion tend to be “network chains” because the actors involved are more likely to be each other’s acquaintances. Thus, the network features linked to such contact chains, besides the actors’ characteristics, also play a major role in understanding how personal moods spread.

As a complex process, the contagion of personal moods often occurs during social interactions when people mimic each other’s voices, postures, facial expressions, or body language^25^. In addition to such physiological responses and processes^6^, as well as the valence of moods and other contextual factors, the extent of mood contagion may vary due to sociodemographic and network features such as gender^26^, power^27,28^ and similarity between the actors^29^ during contacts. Certain events or situations further affect how strongly people express their feelings. Negative events or network ties, in particular, are more likely to draw intense emotional responses than neutral or positive events or ties^5,30^. The mechanisms by which moods spread also differ. For example, happiness or positive affect tends to circulate among network members through a mimicry-based approach. In contrast, anger requires more social appraisal to spread, and negative affect may not be changed by social interaction^21,31^.

### Reconstructing network chains from contact diaries

To capture how viruses and moods spread from contact to contact, researchers have relied on various contact diaries that require participants to record information about all interpersonal contacts on a daily basis. Epidemiologists have used large-scale, short-term (e.g., 24-h) contact diaries to show how infections spread among close contacts^23^. By contrast, longer-term, in-depth contact records have helped to trace how attitudes and behaviors are transmitted among personal contacts^24^. Like social media, in-depth contact diaries yield longitudinal data that links personal moods to social interactions. Unlike social media, however, contact diaries record diary keepers’ self-reported feelings towards each specific social interaction. By covering comprehensive social interactions, the contact diary data enables one to reconstruct network chains through which personal moods spread in everyday life.

Such network chains are particularly critical when a group of network members participate in the same diary study and evaluate the same contact situations from their own perspectives. Using these pooled evaluations, researchers are able to match the self-reported records from interlocking diaries in order to cross-check a pair of actors’ emotional expressions towards each other during each unique contact. As a result, it is feasible to examine how two people can have different perceptions about a shared contact between the pair.

By extending diary studies of social interaction in general, and recent efforts to track all-encompassing interpersonal contacts^10,24,32–34^, we applied the diary approach of social network studies to examine mood contagion along a series of contacts. Of the 171 contact diaries that participants updated for at least 3 and up to 23 months, we analysed mood states of 963 triplets from 113 interlocking diaries by comparing the mood states before and after every contact (Table 1). We define interlocking as occurring when a participant (ego, or A) reported a contact with another participant (degree-1 alter, or B) who had previously interacted with a third participant the ego did not know (degree-2 alter, or C) in the past two days. Because the longitudinal series of interactions among these participants constitute clear chains that also link each other’s acquaintances within the social circles, the resulting network chains differ from regular contact chains that facilitate flu infections, which could consist of total strangers.

**Table 1:** The frequency table of mood states before and after 28,030 contacts reported by the ego of the 963 triplets.

Before	After					
	Very good	Good	Fair	Bad	Very bad	Total
Very good	3156 (98.7%)	40 (1.3%)	0 (0%)	0 (0%)	0 (0%)	3196 (100.0%)
Good	183 (0.9%)	18,844 (97.7%)	231 (1.2%)	21 (0.1%)	2 (0.01%)	19,281 (100.0%)
Fair	30 (0.5%)	841 (15.4%)	4531 (83.0%)	59 (1.1%)	1 (0%)	5462 (100.0%)
Bad	2 (2.4%)	9 (10.7%)	15 (17.9%)	48 (57.1%)	10 (11.9%)	84 (100.0%)
Very bad	0 (0%)	3 (42.9%)	3 (42.9%)	0 (0%)	1 (14.2%)	7 (100.0%)

**Figure 1:**
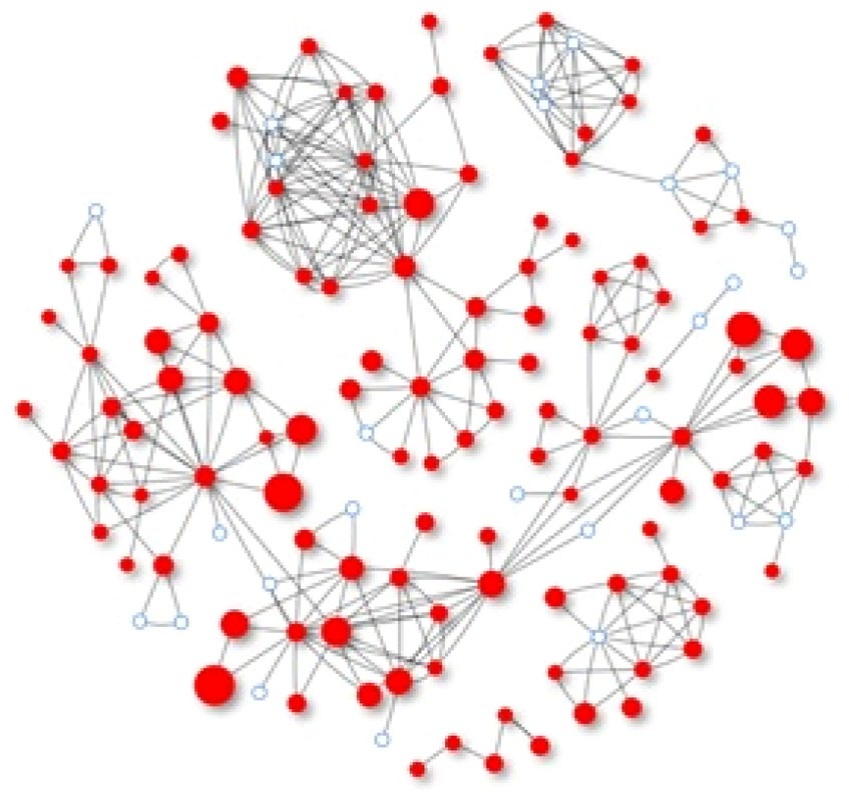
The acquaintance networks of the participants who contributed contact diaries for analysis in the study. The 113 participants who we identify as playing the role of ego appear as red solid nodes. The sizes of the red nodes are proportional to the frequencies in which they appeared as an ego in the 963 triplets of ego, degree-1 alter, and degree-2 alter.

Following such network chains, we examine whether and how personal moods spread first from C to B during an earlier contact, then from B to A during a subsequent contact. To better reveal mood changes from contacts, we used mixed effects logistic regression models to analyse how likely the mood of A will change in the same direction of mood as B experienced in their previous contact with C. The analysis also considers other possible explanatory variables that may influence A’s mood change, including both A’s and B’s mood before the contact, B’s mood change over time from a preceding contact with C to the moment right before the contact with A, as well as covariates such as A’s attributes, A’s relationship with B, and the situations surrounding each contact between A and B (Fig. 1).

## Results

**Figure 2:**
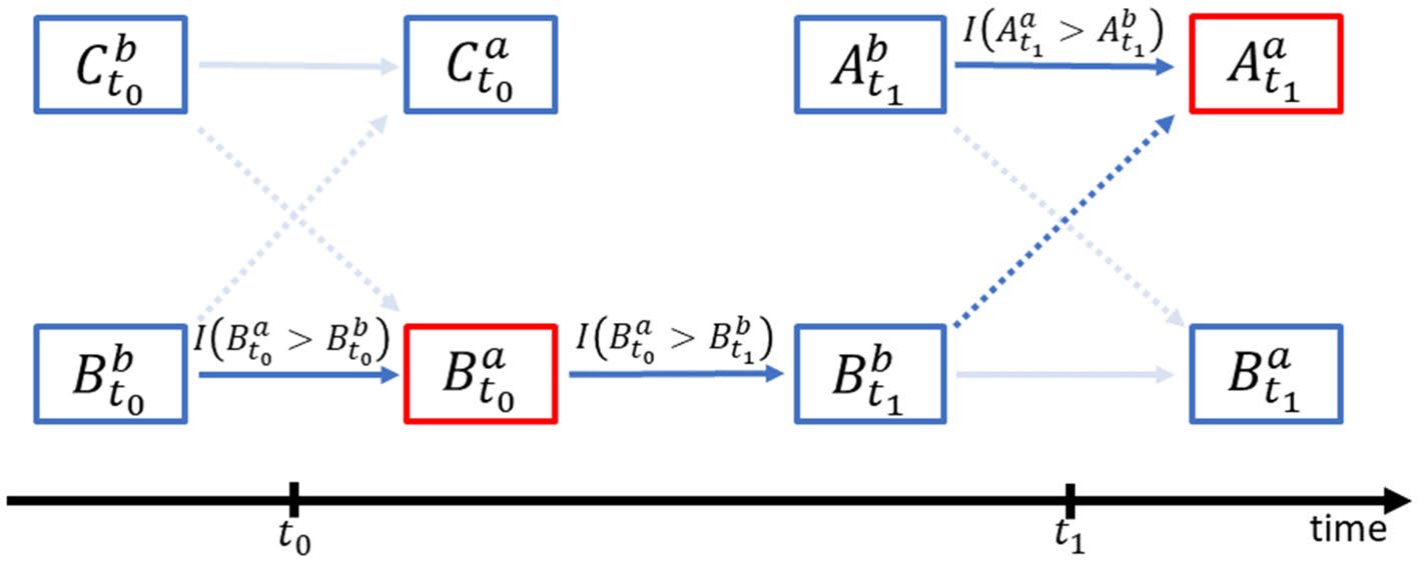
Schematic plot of mood states reported by a triplet of participants A, B and C, in which B and C made a contact at time ${t}_{0}$ before B and A contacted each other at time ${t}_{1}$ within two days. The mood states before and after the contact at time ${t}_{1}$ of participant A are denoted by ${A}_{{t}_{1}}^{b}$ and ${A}_{{t}_{1}}^{a}$, respectively. The possible mood changes of the participants, either due to a social interaction or just because time passed, are presented by indicator functions as they are marked above solid arrows. The dotted arrows indicate the influence of the contact persons.

Table 2 shows the pooled model estimates of factors that helped explain under what circumstances positive personal moods spread from a recent contact with a person to the next contact with another. To better distinguish how personal moods spread from C to B to A, to be more specific, we examined the effects of both A’s and B’s moods at different stages. As shown in Fig. 2, the study’s main goal is to explain whether A’s personal moods improved after interacting with B at time ${t}_{1}$ ${(A}_{{t}_{1}}^{a}$), compared to A’s mood right before the contact (${A}_{{t}_{1}}^{b}$). When the value of ${A}_{{t}_{1}}^{a}$ is greater than ${A}_{{t}_{1}}^{b},$ the value of the response variable equals 1, indicating a positive mood change. The response variable equals 0 if ${A}_{{t}_{1}}^{a}$ is not greater than ${A}_{{t}_{1}}^{b}$.

**Table 2:** Pooled estimates of the coefficients with standard errors and corresponding odds ratios with 95% confidence intervals in the mixed logistic regression model of positive mood change from 100 imputed datasets.

Variable^1^	Coefficient	Std. Error	p value	Odds Ratio	95% C.I
**Measures of mood state**					
A’s mood was good or very good before contact with B	−3.665***	0.143	< 0.001	0.03	(0.02, 0.03)
B’s mood improved during a previous contact with C	0.611***	0.185	< 0.001	1.84	(1.28, 2.65)
B’s mood later worsened since the end of contact with C	−0.037	0.152	0.809	0.96	(0.72, 1.30)
**Ego-level attributes**					
Age	−0.595*	0.274	0.030	0.55	(0.32, 0.94)
Male	−1.683*	0.740	0.023	0.19	(0.04, 0.79)
Openness	−0.116	1.199	0.923	0.89	(0.08, 9.34)
Conscientiousness	−0.860	0.746	0.249	0.42	(0.10, 1.83)
Extraversion	−0.763	0.974	0.433	0.47	(0.07, 3.15)
Agreeableness	0.623	0.812	0.443	1.86	(0.38, 9.16)
Neuroticism	0.261	0.961	0.786	1.30	(0.20, 8.54)
**Alter-level attributes**					
Same sex	−0.379	0.231	0.102	0.68	(0.44, 1.08)
*Relative status (ref: same rank)*					
Alter higher	0.318	0.205	0.120	1.37	(0.92, 2.05)
Alter lower	−1.112**	0.372	0.003	0.33	(0.16, 0.68)
Strong tie	0.052	0.279	0.851	1.05	(0.61, 1.82)
Embeddedness	2.436*	0.954	0.011	1.26	(1.05, 1.54)
Strong tie x Embeddedness	−1.317*	0.664	0.047	0.88	(0.77, 1.00)
**Contact-level attributes**					
*Initiation (ref: by appointment)*					
By ego	−0.057	0.156	0.716	0.94	(0.70, 1.28)
By alter	−0.025	0.170	0.884	0.98	(0.70, 1.36)
By chance	−0.485	0.371	0.191	0.62	(0.30, 1.27)
In person	0.101	0.133	0.446	1.11	(0.85, 1.44)
Other people around	−0.235	0.150	0.116	0.79	(0.59, 1.06)
Longer than an hour	0.795***	0.143	< 0.001	2.21	(1.67, 2.93)
*Parts of the day (ref: morning)*					
Afternoon	0.147	0.132	0.267	1.16	(0.89, 1.50)
Evening and night	0.624***	0.145	< 0.001	1.87	(1.40, 2.48)
*Content (ref: others)*					
Work	0.002	0.171	0.989	1.00	(0.72, 1.40)
Leisure	0.081	0.187	0.666	1.08	(0.75, 1.56)
Social chat	0.165	0.132	0.213	1.18	(0.91, 1.53)
Daily routine	0.064	0.131	0.628	1.07	(0.82, 1.38)
*Instrumental gain (ref: hardly any)*					
A great deal	3.292***	0.228	< 0.001	26.9	(17.2, 42.0)
Somewhat	1.692***	0.148	< 0.001	5.43	(4.06, 7.26)
A loss	0.367	0.516	0.476	1.44	(0.53, 3.97)

^1^All variables are binary except age, for which the odds ratio is calculated based on the increase of ten years, and embeddedness, for which the odds ratio is calculated based on the increase of one-tenth of the embeddedness score.

*: p-value < 0.05; **: p-value < 0.01; ***: p-value < 0.001.

To control for the possible effect of autocorrelation when examining A’s mood change, it is necessary to first consider A’s own mood state before the contact, or ${A}_{{t}_{1}}^{b}$. To distinguish from the “influence” functions (e.g., the direction between B and A) that are shown as dotted lines, these “control” functions (e.g., between ${A}_{{t}_{1}}^{b}$ and ${A}_{{t}_{1}}^{a}$) appear as solid lines in Fig. 2. When A’s mood was either “very good” or “good” before interacting with B, the estimate of parameter ${\beta }_{1}$ in the logistic model (1) dropped to − 3.665 (p < 0.001), as shown in Table 2, suggesting that it was very unlikely that A’s mood could get any better after the contact. The estimated odds ratio (OR) of such further improvement from an already-positive mood was only exp (− 3.665) $=$ 0.03, indicating a substantially lower likelihood, with a 95% confidence interval of (0.02, 0.03), compared to a change for the better mood from either a “fair” or “bad” or “very bad” mood before the contact.

After controlling for A’s preexisting mood state, it becomes clearer whether and how B’s mood changes at two stages may subsequently affect A’s mood change. The first step focuses on the extent to which B’s mood improved from the previous contact with C along the contact chain at an earlier time ${t}_{0}$. This explanatory variable equals 1 if the value of B’s mood after interacting with C ${(B}_{{t}_{0}}^{a})$ is greater than that before the interaction (${B}_{{t}_{0}}^{b}$); and it equals 0 otherwise.

The rationale of this essential explanatory variable lies in the fact that personal mood may “spread” from a third person (C) to the second person (B), then to the first person (A), by means of two consecutive interpersonal contacts, first between C and B, then between B and A. If both the first and the last contacts brought about positive mood changes, then it becomes more convincing to claim that the positive mood at the end of the contact chain may have followed through the series of positive mood changes in the previous steps of the consecutive contacts.

The positive estimate of 0.611 (p < 0.001) for model parameter ${\beta }_{2}$ indicated that when B’s mood improved after interacting with C, the mood of the next person who contacted B within two days, or A, also had a better chance of improving. The contagion effect on such a positive mood change was significantly large, with an estimated OR of exp (0.611) = 1.84 (1.28, 2.65). In other words, A is 84% more likely to feel better after interacting with B if B had a positive contact with C earlier. Finally, if B’s mood worsened within two days or less since finishing the contact with C, B’s contact with A would not bring about any significant positive mood change for A (${\widehat{\beta }}_{3}=-0.037$, p = 0.809).

Following the same analytic strategies while controlling for the identical covariates across ego, alter, and contact levels, Table 3 reveals how the acquaintance network chains led to A’s negative mood change after interacting with B using the logistic regression model (2). Unlike with positive mood changes, if A was already in bad mood state before interacting with B, that mood could become even worse after contact with B, with parameter estimate ${\widehat{\beta }}_{1}=$ 1.126 (p = 0.005) and OR = 3.08 (1.42, 6.71). The estimated odds ratio suggests that A’s own negative mood prior to interacting with B is three times as likely to linger after the contact.

**Table 3:** Pooled estimates of the coefficients with standard errors and corresponding odds ratios with 95% confidence intervals in the mixed logistic regression model of negative mood change from 100 imputed datasets.

Variable^1^	Coefficient	Std. Error	p value	Odds Ratio	95% C.I
**Measures of mood state**					
A’s mood was bad or very bad before contact with B	1.126**	0.397	0.005	3.08	(1.42, 6.71)
B’s mood worsened during a previous contact with C	0.613	0.328	0.062	1.85	(0.97, 3.51)
B’s mood improved since the end of contact with C	− 0.625*	0.257	0.015	0.54	(0.32, 0.89)
**Ego-level attributes**					
Age	− 0.690*	0.311	0.026	0.50	(0.27, 0.92)
Male	− 0.843	0.841	0.316	0.43	(0.08, 2.24)
Openness	− 1.513	1.449	0.296	0.22	(0.01, 3.77)
Conscientiousness	− 1.294	0.878	0.141	0.27	(0.05, 1.53)
Extraversion	− 2.904*	1.340	0.030	0.05	(0.00, 0.76)
Agreeableness	1.281	0.933	0.170	3.60	(0.58, 22.4)
Neuroticism	− 0.320	1.134	0.778	0.73	(0.08, 6.70)
**Alter-level attributes**					
Same sex	− 0.381	0.301	0.205	0.68	(0.38, 1.23)
*Relative status (ref: same rank)*					
Alter higher	− 0.268	0.275	0.329	0.76	(0.45, 1.31)
Alter lower	− 0.933*	0.425	0.028	0.39	(0.17, 0.90)
Strong tie	1.338	0.827	0.106	3.81	(0.75, 19.3)
Embeddedness	2.002	1.238	0.106	1.22	(0.95, 1.56)
Strong tie x Embeddedness	− 1.234	1.089	0.257	0.88	(0.71, 1.09)
**Contact-level attributes**					
*Initiation (ref: by appointment)*					
By ego	0.761***	0.160	< 0.001	2.14	(1.56, 2.93)
By alter	0.061	0.212	0.774	1.06	(0.70, 1.61)
By chance	0.534	0.432	0.217	1.71	(0.73, 3.98)
In person	0.114	0.151	0.451	1.12	(0.83, 1.51)
Other people around	− 0.088	0.204	0.667	0.92	(0.61, 1.37)
Longer than an hour	0.416*	0.162	0.010	1.52	(1.10, 2.08)
*Parts of the day (ref: morning)*					
Afternoon	0.115	0.183	0.530	1.12	(0.78, 1.61)
Evening and night	0.119	0.187	0.526	1.13	(0.78, 1.63)
*Content (ref: others)*					
Work	0.817***	0.191	< 0.001	2.26	(1.56, 3.29)
Leisure	0.340	0.226	0.133	1.40	(0.90, 2.19)
Social chat	− 0.553***	0.174	0.001	0.58	(0.41, 0.81)
Daily routine	− 0.808***	0.184	< 0.001	0.45	(0.31, 0.64)
*Instrumental gain (ref: hardly any)*					
A great deal	− 0.132	0.408	0.746	0.88	(0.39, 1.95)
Somewhat	− 0.365*	0.177	0.039	0.69	(0.49, 0.98)
A loss	2.494***	0.348	< 0.001	12.11	(6.12, 23.9)

^1^All variables are binary except age, for which the odds ratio is calculated based on the increase of ten years, and embeddedness, for which the odds ratio is calculated based on the increase of one-tenth of the embeddedness score.

*: p-value < 0.05; **: p-value < 0.01; ***: p-value < 0.001.

Similar to the case with positive mood changes, A’s mood is more likely to get worse after interacting with B if B’s own mood deteriorated during a contact with C, but the effect was not statistically significant. If B’s mood had improved since finishing a previous contact with C, however, the chance for A to experience negative mood changes from interacting with B becomes significantly lower (${\widehat{\beta }}_{3}=-0.625$, p < 0.05), with OR = 0.54 (0.32, 0.89). More precisely, the likelihood for the negative mood to spread is reduced by nearly half.

It is revealing to compare how these personal moods during and after earlier contacts led to A’s positive and negative mood changes. First, A’s mood before the contact affects A’s mood after the contact. When A already felt good before interacting with B, there was no room to improve further along the fixed scale of mood states (the odds ratio is near zero). If A felt bad before the contact, however, it was three times more likely that A would experience a worse mood after the contact.

In other words, both positive and negative personal moods tended to spread along network chains, but the intensity of the contagion effects differed between the two. When B experienced a positive mood change from an earlier contact with C at time ${t}_{0}$, A was 84% more likely to also have such a positive change from a contact with B at time ${t}_{1}$, and the increase is statistically significant at the 0.001 level (Table 2). Likewise, if B’s mood changed for the worse from an earlier contact with C at time ${t}_{0}$, A was also 85% more likely to have a negative mood change at time ${t}_{1}$ (Table 3). Although the percentage increase matches that of the positive mood contagion, the contagion effect of negative moods is only barely significant (p = 0.062).

It becomes more intriguing to trace A’s mood changes back through a network chain: from A to B to C, or more precisely, from a contact between A and B at time ${t}_{1}$ backward to a previous contact between B and C at time ${t}_{0}$. For A’s positive mood change at time ${t}_{1}$, B’s negative mood change from time ${t}_{0}$ to time ${t}_{1}$ may not matter much. B’s positive mood change from time ${t}_{0}$ to time ${t}_{1}$, in contrast, tended to reduce the possibility that A’s mood changed for the worse.

The contagion effects of personal moods are noteworthy as they remain significant and substantial while the models also include key covariates at ego, alter/tie, and contact levels. For example, while ego’s age, gender, and extrovert personality all help distinguish who are more likely to experience positive or negative moods, the changes in such moods at different stages of the network chain continue to show signs of transmission independent of the effects of the sociodemographic factors (Tables 2 and 3). Ego’s personal moods after a contact with the alter also vary by the alter’s status relative to ego’s, and by the degree of the alter’s embeddedness in ego’s network—or how extensively the alter knows ego’s other network members. The extent of mood changes further varies depending on various contact situations, particularly how long a contact lasted, who initiated the contact, what part of the day the contact took place (only positive mood change is more significant during the evening or night), whether the contact was related to work, a social interaction, or a daily routine, and how much the contact benefited the ego. In addition to these separate effects directly related to ego, alter, and contact, the analysis indicates that different actors’ personal moods may have indeed spread along a series of interpersonal contacts.

## Discussion

To reiterate the overall findings, personal moods spread along a chain of social interactions in the same way that a virus transmits from one person to another. Unlike virus transmission, however, positive and negative moods differ in how they spread along the chain. When one’s mood improves during a previous contact with a third person, that mood change tends to be highly contagious in the subsequent contact. If the mood later worsens since the previous contact ended, however, that degraded mood state has no significant impact on the other person’s mood during the subsequent contact.

If one’s mood worsened during the previous contact, this bad mood is more likely to spread to the next contact, although to a lesser degree. But when the previous negative mood reverses back to a better mood before the next contact starts, the mood’s contagion effect from that latter contact becomes much less likely. Furthermore, bad moods stop spreading when the second person in the three-person contact chain regains a better mood (thus becoming non-contagious) before making contact with another person. This broken chain of negative mood contagion resembles that of disease transmission, as a virus stops spreading when an infected person recovers from the earlier infection.

Unlike disease transmission, therefore, mood contagion may follow different paths depending on the valence of moods. By integrating the concept of disease transmission into the diary design, the current study on diverse contagion flows also extends earlier studies to new directions. While previous studies examined how emotions may spread from person to person or along a series of interpersonal ties^21,22,32^, our design uses a bottom-up approach to capture different chains of “contacts”, a level underneath the ties. Such a bottom-up approach echoes the “day reconstruction method” or experience-sampling techniques used to sum up one’s activities and emotions from a series of episodes in daily life^35^, and attempts to move a step further.

By examining emotions at different moments in everyday life, recent studies have shown that older adults are more emotionally stable and better at emotion regulation that boosts psychological well-being^36–38^. By linking each moment of social interaction and tracing how moods transit along the contact chains, the current study further indicates that older people are less likely to be influenced through contact with others, in terms of both positive and negative moods. For conventional network studies, the extent of mood contagion would vary by the characteristics of both individuals and interpersonal ties. When we divide each tie into a number of contacts, the mechanisms of mood contagion would not only depend on the features at both individual and tie levels but also vary from contact to contact.

In addition to differentiating the valence of personal moods into positive and negative, it is also important to note how the nature of emotion contagion differs from disease infection. While one can spot and infer disease infection by checking the onset of certain influenza-like symptoms after a person has been in contact with those who have had similar symptoms, it could be difficult to identify the exact source of such an infection if the incubation period is long or the patient had contacted different people with similar symptoms. Unlike most disease infections, one’s personal mood may spread to other persons instantly, and the effects may last for seconds or hours. Temporality is therefore one of the most challenging issues that arises when studying mood contagion.

Because most preexisting studies on mood contagion lack solid data with a time dimension, it has been difficult to identify any clear pattern that shows how personal moods transit from a person to another^33^. Instead, some studies have explored “concurrent moods” using similar research designs^10^. Having learned from the limitations of previous studies, the current study adjusted the diary platform and recruiting strategy in an effort to collect interlocking contact diaries that helped us construct a clear temporal data structure of mood contagion (as in Fig. 2).

Such a temporal sequence becomes feasible when there are a large number of connected participants that enable researchers to match mutual evaluations of personal moods towards the identical contacts and to link consecutive interpersonal contacts that occur within two days. Even though it would have been impractical to ask participants to record every contact and every mood change at every minute, we managed to link each mood state with one of three different time slots each day: morning, afternoon, and evening. In addition, we excluded other subsequent contacts during the same time slot to avoid any confounding “concurrent moods”. With such a sequential data structure, it became possible to reveal the patterns of mood contagion by clearly identifying how personal moods move from an “affected” person to the next person along the network chain.

Rather than analyzing just one mood state at a specific point of time, our research thus contributes to the studies on mood contagion by showing how personal moods change over time. For epidemiologists, a virus infection is likely when a person starts to show influenza-like symptoms after making close contact with a flu patient. Similarly, mood contagion is plausible when one’s mood changes from a certain state to another—from good to very good, from good to bad, and so forth after an interaction. By incorporating such a prerequisite into our research design, we show that mood contagion only occurs when one’s mood changes. We believe that such a design could help clarify the precise chains of mood contagion, which have been extremely difficult to document.

Some limitations are noteworthy. As with other in-depth diary studies^39–42^, our sample of participants is not representative of any population^10,24^. The volunteer participants in our study skew toward being young, female, and better educated, who tend to be more competent and comfortable in keeping tedious contact records^24^. Future studies could benefit by comparing the findings from this subgroup to that from a group of participants with different ages, sociodemographic features, and educational backgrounds.

Another limitation is the missing information about how some diary keepers felt, and how other keepers believed the other person felt, before and after the pair interacted with each other. To obtain reasonably comprehensive contact data while trying to retain some volunteers in the study, we encouraged the participants to record their daily contacts two or three days a week for at least three months. Thus in some of the interactions that A recorded occurring between A and B, B did not report the same contact, and so on, which causes incomplete information about self-reported mood states. Consequently, the total number of triplets of A, B, C who were linked in a network chain and who self-reported all their mood states was too small for modeling mood contagion. To fill such gaps, we used imputation methods that incorporated both other-reported mood states and previous self-reported mood states. The imputation allowed us to model mood contagion by compiling sufficient triplets of participants linked in a network chain.

While similar research shows that it was reliable to substitute self-reported mood scores with other-reported mood states, which reached an accuracy rate of 99.0% within a 1-point difference^10,49^, the current study took the imputation a step further by including one’s own self-reported mood states from other interactions during the study period as another base to estimate. As with most other studies of self-reported attitudes and emotions^10,40,42^, such missing information could lead to unknown biases despite a detailed imputation design. For the current study in particular, one might have changed the patterns of self-reporting mood states over time due to various uncontrollable circumstances. It would be revealing to explore whether and how such circumstances have affected the patterns of self-reported mood states. More fittingly, it is desirable to alleviate the need for imputations in future studies in which both parties of the same interaction report their mood states simultaneously.

One more limitation concerns the sample of contacts. As indicated in earlier diary studies^24^, it is infeasible to ask a group of participants to record all interactions they experience for months. To collect a reasonable number of reliable contact records, our research design thus allowed participants to skip writing in their diaries on the days when they were too overwhelmed to record the contacts they had made. As a result, the contact records we analysed may not be comprehensive, and the hidden factors in these missing chains might also affect subsequent mood changes. In particular, a missing contact that occurred in between the contact between C and B at time ${t}_{0}$ and the contact between B and A at a later time ${t}_{1}$ might have rendered unknown effects on B’s mood prior to the latter contact. Future studies about mood contagion should attempt to address ways to invent these gaps in collecting acquaintance network data.

We originally asked the participants to assess the mood through a 5-point Likert scale ranging from "very bad" to "very good," then transformed this numeric data to binary in analyses. An alternative method would be to take the difference of A’s emotion before and after the interaction with B as the dependent variable. Similarly, we could have measured all three mood states in Tables 2 and 3 as numeric: A’s mood before contact with B, B’s mood change during a previous contact with C, and B’s mood change since the end of contact with C, and combined the results from the two tables in a single regression analysis.

The dependent variable defined as the difference of A’s moods before and after interacting with B would contain three categories: positive change, negative change, and unchanged. Instead of the two separate logistic models for positive change and negative change, we could then use one trinomial model to regress the dependent variable on B’s mood, A’s attributes, alter-level and contact-level attributes. In doing so we would assume that these attributes shared the same effects on both positive change and negative change of A’s mood after contact with B. As shown in Tables 2 and 3, a few attributes nonetheless exert different effects on positive and negative mood changes. In order to identify such crucial differential effects that vary by valence, it is more revealing to use binary transformation and perform two separate logistic analyses.

The deliberate design of the diary platform captured in-depth interconnected contact records that enabled us to identify clear flows of mood contagion along network chains in everyday life. Such contact records facilitate the temporal analysis of mood contagion by integrating the strengths of both studies of egocentric networks, which examine social connections from a focal person’s perspective, and that of complete networks, which focus on the structural features of social connections typically within well-defined groups. The analysis of the dynamic mood contagion further incorporates unique yet intertwined features at ego, alter and contact levels. By virtue of both structural and temporal perspectives, the current study helps uncover the mechanisms under which positive and negative mood changes spread along contact chains that in some important ways resemble yet differ from the typical flow of disease infections.

To further advance contributions to the literature, it is plausible to add the strengths of emotion studies to the research on contact diaries in general, and that on mood contagion in particular. For example, future studies could adopt more fine-grained and validated measures of moods, preferably some abridged versions of the Multidimensional Mood State Questionnaire (MDMQ)^43,44^. Another natural extension would be expanding the dimensions of mood to explore the extent to which varying degrees of arousal (such as excitement, anger, or sadness) may trigger diverse patterns of mood contagion^45–47^. Even though the current paper has shown the temporal sequence of mood contagion by combining different perspectives of social network studies, incorporating more refined measures and comprehensive angles of personal moods from yet another field could further enhance the potentials brought by more interdisciplinary efforts.

## Methods

### Ethics

This study was approved by the Institutional Review Board (IRB) on Humanities and Social Science Research, Academia Sinica (AS-IRB-HS 02-16039). To protect personal privacy, we have removed all personal identifiers from the data and assigned each participant as well as each contacted person a unique identification number. This study was performed in accordance with the declaration of Helsinki and followed by the approved protocol. The informed consent was obtained from all subjects via our online platform.

### Data collection and the content of diaries

In September 2017, we started inviting adult participants in Taiwan who had provided high quality data in an earlier study using contact diaries, in addition to recruiting other participants by snowball sampling. By the end of July 2019, 171 participants had completed recording diaries for at least 10 days a month, for a minimum of 3 months, or had recorded more than 5 contacts a day for at least 5 days a month.

In order to reconstruct interlocking diary data from multiple sources, we encouraged the participants to invite their network members to also participate in the study. When any two participants reported or evaluated their perceptions of an identical contact between the pair, these overlapping diaries help determine the extent to which the records from both sides matched or deviated from each other, thus providing a more solid criterion of data validity.

The diary platform asked all participants to first watch an instructional video about how to record the contact diary and how to invite other participants. To ensure the quality of the diary data, the research team checked the validity of all diary entries from each participant weekly. When we suspected and verified that a specific participant had not been keeping the diary properly, the participant was put on our alert list, and any data entered by the participant was excluded from further analysis. We also performed quality checks on the numbers of contact persons (alters) and the answers on alter-alter ties. To encourage participants to maintain quality diary keeping throughout the study period, as well as to compensate for the participants’ loss of time, we conducted a lottery drawing weekly. By assigning different weights to the lottery system based on the quality and quantity of each participant’s diary records during the week, the drawing gave higher chances for winning more-favorable monetary rewards to those who had spent more time and effort on their diary keeping.

We determined whether a participant contacted another participant by checking their unique “membership ID” numbers (or “ego ID” in our data file). Our diary platform assigned a sequential number to each participant who first registered before answering questions in the diary. As a diary keeper (ego) entered a contact record, the system saved the personal information about each contact person (alter) and compiled a separate list of alters for each ego. For subsequent contacts with an alter already listed, ego just selected the alter’s name from the existing list and entered the information about contact features. When contacting someone not in the alter list, ego added the person as a new alter to the platform, so the list kept growing.

The unique participant IDs stored in the comprehensive alter lists enabled us to link all participants and differentiate the roles they play in the overlapped networks, further aided by our referral design. We sent a message to all participants who registered and recorded their contact records that had their ID embedded in a link, and encouraged them to forward the link to fellow participants in their alter lists and to other friends and relatives so more network members could participate in the same diary study. This referral process allowed us to follow three guidelines to identify whether and how the participants actually contacted one another.

First, when an invitee (person A) used a forwarded link to register, the platform first added the participant (person B) who forwarded the link, marked with person B’s ID, to person A’s alter list; it then recorded A’s ID back to B’s alter list. Second, if a registered participant (person A) received a forwarded link from another participant (person B) who was in A’s preexisting alter list, A would select B from A’s alter list to record this specific contact. The forwarding then linked the two IDs and confirmed the connection between A and B. Third, if a registered participant (person A) received a forwarded link from another participant (person B), who was not in A’s alter list and who differed from the person that originally got A registered, B was added to A’s alter list and both A’s and B’s IDs were linked in the database. In sum, the referral design helped us to use unique IDs to identify who contacted who across “interlocking diaries,” and assure whether a certain “alter” in person A’s diary was the “ego” of person B’s diary, and so on.

The contact diaries, along with an accompanying survey questionnaire on sociodemographic characteristics, yield versatile data nested at three levels. At the diary keeper’s (or ego’s) level, we used the following variables to highlight the individual’s demographic background and personality: age groups, gender, and Big Five personality traits—openness, conscientiousness, extraversion, agreeableness, neuroticism (OCEAN)^48^. The attributes at the alter level (or the ego-alter tie level) include alter’s gender, status ranking relative to ego, and tie strength with ego. To capture an alter’s position in ego’s networks, we followed the latest studies to further construct a structural measure of each alter’s embeddedness^49^, which refers to the degree to which an alter was familiar with each of ego’s other degree-1 alters.

Features at the contact level contain the main variables of interest in this study and other control variables. For each contact, the participant (ego) reported personal moods before and after interacting with a degree-1 alter by selecting one of the following response categories: (1) very bad, (2) bad, (3) fair, (4) good, or (5) very good. With the same criteria, the ego also evaluated each degree-1 alter’s mood before and after the identical contact. Other contact details included who initiated the contact, the mode of interaction (i.e., in-person, video, voice-based messages, or text-based messages only), whether there were other people also present during the interaction, duration, when the contact took place (i.e., morning, afternoon, or evening), the context of the contact (i.e., work, leisure, social chat, and daily routine), as well as the degree of ego’s instrumental (or substantive) gain (or loss) from the contact (refer to Supplement A for the full content of the diary). Each record in the contact diary represented an episode of one-on-one contact. If the participant met many people at the same time, they recorded only the one-on-one interactions that involved at least three sentences of either verbal or text-based exchanges.

Because the sampling schema of “interactions” affects the time resolution for the analysis of mood contagion, we were particularly cautious regarding this critical step by weighing the tradeoffs between the time resolution required by the temporal analysis and the feasibility of diary keeping by the participants. The participants recorded contact diaries voluntarily. To have a better time resolution for tracking mood contagion, we encouraged participants to record all one-on-one contacts as comprehensively as possible on the days when they kept the diary. For each contact, the participants reported whether it occurred (or started) mainly during the (1) morning, (2) afternoon, or (3) evening/night, which enabled us to align different contacts along a clear temporal sequence, though the broad time frame prevented us from examining mood contagion utilizing a finer time resolution (by hour or minute, for example). Consequently, we considered the longest time lag between B’s contact with C and B’s contact with A to be two days for modeling mood contagion from C to B to A. For example, we retrieved diaries of participants A-B-C in which contact between A and B occurred in the evening and contact between B and C was in the afternoon or the morning of the same day, and the evening or the afternoon of the previous day.

### Retrieving data for modeling

Because some of the participants knew each other before the study, the contact diaries we collected were partly overlapped or interlocked, thus revealing both ego-reported and alter-reported personal moods of identical interactions. However, sometimes a participant (ego) recorded a contact with an alter who either did not record the same contact because they skipped recording in their diary on that day or who was not a study participant. In both types of case we were unable to cross-check the diary data for mutually perceived moods. To analyse mood contagion more precisely, we retrieved information about contacts between egos and degree-1 alters who were both study participants as well as between degree-1 alters and degree-2 alters within the past two days.

To better examine how personal moods spread among network members, we constructed our database only from contacts between a pair of participants who knew each other personally. In our diary study between 2017 and 2019, a total of 171 participants recorded their “before and after” moods on 313,347 one-on-one contacts. To study whether the mood change of participant B after contact with participant C would affect the mood change of B’s next contact person, A, we first identified pairs of participant A, who played as an ego, and participant B, who played as A’s alter. By excluding 42 participants who did not record any contact with any other participants, we kept 129 participants who had contact with each other at least once during the study period. As explained earlier, someone who plays an ego’s role may be another ego’s alter. After further removing participants who recorded fewer than 20 contacts in the diary, we finally retained 113 participants who played as an ego with at least one other participant playing as one of the alters. We then tracked whether B also had contact with yet other participants (other than A) within the past two days. Because an ego may have interacted with more than one participant (alter), we were able to identify 263 ego-alter pairs from the 113 egos. Furthermore, each alter of A’s in the pairs (or B, while playing an ego’s role themselves) may also have contact with more than one participant other than A (or C, playing as B’s alter) within two days. Step by step, we finally reconstructed a total of 963 triplets (A, B, C) from 113 interlocking contact diaries, which contained 56,060 contacts (refer to Supplement B for the flow of retrieving data).

In other words, we were able to identify 263 pairs of participants, one as ego (A) and the other as degree-1 alter (B). In addition, B must have contacted at least one other acquainted participant in the past two days who A did not know (that is, C [who would be A’s degree-2 alter]). As a result, we were able to compile a total of 963 triplets of participants who were linked in a network chain (from A to B to C, or from ego to a degree-1 alter to a degree-2 alter) out of all possible ties extending from 113 participants who ever played an ego’s role in such a network chain. Figure 1 maps the acquaintance networks of these 113 participants who appeared at least once in such a triplet.

To highlight their roles in the network chains, we used red solid nodes to identify the 113 participants who were ever the ego in a contact chain in Fig. 1. In contrast, we used white (or blank) nodes to represent other participants who were either degree-1 or degree-2 alters in the triplets we analyse. While the red nodes could be either an ego, degree-1 alter, or degree-2 alter, the white nodes were other participants who acted only as degree-1 or degree-2 alters in these triplets. It is noteworthy that we limited our data analysis to the pairs of participants along a network chain who knew each other. That is, we only analysed the network chains in which ego and degree-1 alter (or A and B) knew each other, as did degree-1 alter and degree-2 alter (or B and C). To avoid potential bias due to possible direct influences from degree-2 alters to egos, we excluded the chains in which ego and degree-2 alter (or A and C) also knew each other. This exclusion further justifies our usage of “acquaintance network chains”, which involve two directed ties, from C to B to A, through which personal moods may transmit within the specified time frame.

The acquaintance networks in Fig. 1 consist of five components or subsets, including a very large one that stretches from left to right, and a very small one of only 5 participants, on the bottom. The size of each red node is proportional to the number of times (or the number of contacts) when the participant was the ego of the 963 triplets. The smallest and largest nodes correspond to 1 and 28 contacts, respectively, while the median node size is 7 contacts.

Among the 963 triplets of participants, 113 unique egos (A) reported personal moods in a total of 28,030 contacts with degree-1 alters (B). In 72.8% of these contacts, B did not report such moods or other contact situations. Of all the 28,030 contacts between B and degree-2 alters (C), B missed reporting 52.1% of the contacts and C did not report 48.9% of the cases.

During subsequent analyses and modeling, such missing data along the network chains presented a significant challenge to our methodology and interpretations. To diminish the loss due to such missing self-reported mood states, we proposed the following scheme to impute from the known data. First, we retrieved the information about the contacts in which both participants of the pair, say X and Y, reported their own and each other’s mood states. We then summarized the pairs of Y’s mood states, one self-reported by Y and the other judged by X, in a 2 × 2 frequency table. In notation, let ${y}_{ij}$ denote the frequency of contacts, in which Y’s self-reported mood state is *i,* and Y’s mood judged by X is *j*.

The mood state is represented by the numbers from 1 (very bad), 2 (bad), 3 (fair), 4 (good), to 5 (very good). For a mood state that X judged, or *j*, we calculated the mode of Y’s multi-time self-reported mood states based on the observed five frequencies ${y}_{1j},\dots {y}_{5j}$, and denoted the mode as ${m}_{j}$ for $j=1,\dots ,5.$ When Y’s self-reported mood state was missing from a contact with X, but X had rated Y’s mood state as $k$ for the contact, we imputed the missing self-reported mood state of Y’s with ${m}_{k}$. When the mode ${m}_{j}$ is unavailable due to ${y}_{1j}=\dots ={y}_{5j}=0$, we assigned it with one of other modes that were available with equal probabilities. In the event that the highest value of the $\{{y}_{1j},\cdots ,{y}_{5j}\}$ is not unique, we gave a random mode ${m}_{j}$ by one of the most frequently reported mood states. For our logistic regression models, we repeated the above procedures to generate 100 datasets that contain full information about the self-reported mood states involved in the contacts of the 963 triplets.

### Final sample for analysis

Among all participants who played the role of ego in the interlocking diaries, 69% were female (N = 78) and 76% (N = 86) were between the ages of 21 and 50, with an average of 41.6 (S.D. = 13.7). Among all ego-alter pairs, 69.2% were of the same gender. The alter was higher than the ego in terms of relative hierarchical roles (for example, when the alter is a supervisor to the ego) in 17.9% of the pairs, and lower in 26.2% of the cases. About 83.7% of these ego-alter ties were strong (i.e., ego knew alter “very well” according to the structured diaries the participants kept). The embeddedness scores of the alters in the ego’s network ranged from 0 to 0.96, with a median of 0.35 and the first and third quartiles of 0.19 and 0.64, respectively.

By aggregating from the entries below and above the main diagonal in Table 1, which summarized ego’s reported mood states before and after contacts with degree-1 alters, we identified 1,087 (3.9%) of the 28,030 contacts that brought about positive mood changes for the ego, and 364 (1.3%) contacts that resulted in negative mood changes. Of all contacts, the ego reported a total of 22,577 (80.2%) “good” and “very good” mood states before contacts, while only 91 (0.3%) of all contacts were either “bad” or “very bad”. When the mood was good before contact, only 0.9% of such contacts yielded “very good” moods afterwards. When the mood was bad before contact, the chance for such a mood to worsen to “very bad” after contact was 11.9%. Overall, the likelihood that the egos would report being in a better rather than a worse mood after contact with alters was much greater.

After interacting with degree-2 alters within the 963 triplets, degree-1 alters reported positive mood changes in 6.2% of contacts, and negative mood changes in 2.6% of the cases. Compared to their personal moods just after the previous contacts with degree-2 alters, which could occur up to two days earlier, degree-1 alters reported worse moods the moment before interacting with ego in 16.3% of all relevant cases, and better moods in 10.4% of the cases.

Of all 28,030 contacts between ego and degree-1 alter, the majority were either ego-initiated (44.8%) or alter-initiated (35.4%). Nearly half (48.2%) of all contacts were in person, and more than one third (36.0%) lasted longer than an hour. While many contacts encompassed multiple domains in everyday life, about 62.2% involved social chat, close to half (49.1%) were part of daily routines, and only 16.7% were work-related. As with personal moods, another major outcome of such social interactions also varied widely from contact to contact: egos believed that they benefited from contacts “a great deal” (10.3%), “somewhat” (61.0%) or “hardly at all” (27.5%).

### Performance evaluation of imputation method

To evaluate the extent to which the proposed imputation scheme estimated the missing mood status accurately, we randomly partitioned 18,076 contacts, in which both participants reported their own and each other’s moods, into two equal halves. One half served as a training set, and the other half as a test set. We first assigned one of the two mood states for the identical contacts in the test set as missing, then applied the imputation scheme to the training set in order to predict the missing mood states in the test set using the imputation results from the training set.

This evaluation method helped us calculate the difference between the true and the imputed mood scores. After repeating the same procedure 10,000 times, the imputation showed that, on average, 89.1% of the differences were zero, 4.5% negative 1 and 5.5% positive 1, 0.5% negative 2 and 0.4% positive 2. For another robustness check, we also took 30% of the data as a training set and the other 70% as a test set. The results were similar with 88.4% of zero difference and 99.0% within 1. The results of such robustness checks help verify the validity of our imputation method regarding the missing values.

### Statistical analysis

Given an imputed dataset with full information about the self-reported mood state, along with the attributes at ego, alter and contact levels, we examined the effects of mood contagion on positive moods and negative moods separately in two models. For each triplet of ego, degree-1 alter and degree-2 alter (or A, B, C), we denoted $${(A}_{{t}_{1}}^{b},$$ $${A}_{{t}_{1}}^{a})$$, $${(B}_{{t}_{1}}^{b},$$ $${B}_{{t}_{1}}^{a})$$ to be A’s and B’s self-reported mood states before and after the contact at time $${t}_{1}$$, respectively. Similarly, the self-reported mood states of B and C for the contact at an early time $${t}_{0}$$, which was at most two days earlier than $${t}_{1}$$, were denoted by $${(B}_{{t}_{0}}^{b},$$ $${B}_{{t}_{0}}^{a})$$, $${(C}_{{t}_{0}}^{b},$$ $${C}_{{t}_{0}}^{a})$$.

In the case of positive mood changes, we designated the binary variable $I\left({A}_{{t}_{1}}^{a}{>A}_{{t}_{1}}^{b}\right)$ as 1 when A’s mood changed for the better after contact with B at time ${t}_{1}$, and as 0 otherwise. As depicted in Fig. 2, the after-contact mood state ${A}_{{t}_{1}}^{a}$ could be directly influenced by both ${A}_{{t}_{1}}^{b} \mathrm{and} {B}_{{t}_{1}}^{b}$, the two mood states of A and B before the contact at time ${t}_{1}$, and indirectly affected by ${B}_{{t}_{0}}^{a},$ or B’s mood state after a contact with C at time ${t}_{0}$. Similarly, the mood state ${B}_{{t}_{0}}^{a}$ could be influenced by ${B}_{{t}_{0}}^{b}$. To examine the indirect effect of ${B}_{{t}_{0}}^{a}$ on ${A}_{{t}_{1}}^{a}$, we defined two binary variables $I({B}_{{t}_{0}}^{a}{>B}_{{t}_{0}}^{b})$ and $I({{B}_{{t}_{0}}^{a}>B}_{{t}_{1}}^{b})$ as labelled in Fig. 2, indicating whether B had a positive mood change right after a contact with C at time ${t}_{0}$ and whether B’s mood deteriorated before interacting with A, respectively. To further adjust for the potential direct effect of ${A}_{{t}_{1}}^{b}$ on A’s mood change, we defined the variable $I\left({A}_{{t}_{1}}^{b}>3\right)$ indicating whether A’s mood before the contact with B was either good or very good.

We then proposed the mixed effects logistic regression model for modeling the positive mood change as:

1 $${\text{logit}}\left( {E\left( {I\left( {A_{{t_{1} }}^{a} > A_{{t_{1} }}^{b} } \right){ }} \right)} \right) = \beta_{0} + \beta_{1} I\left( {A_{{t_{1} }}^{b} > 3} \right) + \beta_{2} I\left( {B_{{t_{0} }}^{a} > B_{{t_{0} }}^{b} } \right) + \beta_{3} I\left( {B_{{t_{0} }}^{a} > B_{{t_{1} }}^{b} } \right) + \mathop \sum \limits_{l = 1}^{p} \gamma_{l} X_{l} + a_{A} ,$$

where *p* covariates $X_{l}$’s include attributes at ego, alter, and contact levels to adjust for potential confounding effects, and the random component ${a}_{A}$ is a normal distribution with a mean of zero and a constant variance for modeling the variation of unmeasured factors among the 113 participants.

The left-hand side of the logistic model (Eq. 1) represents the odds in log scale that A will have a positive mood after contact with B, given that the covariates and coefficients on the right-hand side are known. To measure the effect of one covariate of interest on the log odds of A’s positive mood change, we can calculate the difference of the log odds between two specific levels of the covariate while holding the levels of the other covariates fixed. For example, if the covariate of interest is the outcome of B’s mood change in a previous contact, i.e., $I\left({B}_{{t}_{0}}^{a}{>B}_{{t}_{0}}^{b}\right)=1 \mathrm{\,or\,} 0,$ the difference of the log odds turns out to be the coefficient ${\beta }_{2}$ in Eq. (1). In other words, the exponential function of the coefficient, $\mathrm{exp}({\beta }_{2})$, is the odds ratio that A will have a positive mood after contact with B given that B had a positive mood change in the previous contact, compared to B does not experience the positive mood change.

Similarly, we defined the response variable $I({A}_{{t}_{1}}^{a}{<A}_{{t}_{1}}^{b})$ for negative mood change, which indicates whether A changed mood for the worse after interacting with B at time ${t}_{1}$. The three main corresponding independent variables are $I\left({B}_{{t}_{0}}^{a}{<B}_{{t}_{0}}^{b}\right),$ $I({{B}_{{t}_{0}}^{a}<B}_{{t}_{1}}^{b})$ and $I\left({A}_{{t}_{1}}^{b}<3\right)$, respectively, indicating whether B’s mood changed for the worse during the previous contact with C at time ${t}_{0}$, whether B’s mood before contact with A improved from the mood state after the contact with C, and whether A’s mood before the contact with B was bad or very bad. The mixed effects logistic regression model for the negative mood change is then:

2 $${\text{logit}}\left( {E\left( {I\left( {A_{{t_{1} }}^{a} < A_{{t_{1} }}^{b} } \right){ }} \right)} \right) = \beta_{0} + \beta_{1} I\left( {A_{{t_{1} }}^{b} < 3} \right) + \beta_{2} I\left( {B_{{t_{0} }}^{a} < B_{{t_{0} }}^{b} } \right) + \beta_{3} I\left( {B_{{t_{0} }}^{a} < B_{{t_{1} }}^{b} } \right) + \mathop \sum \limits_{l = 1}^{q} \gamma_{l} X_{l} + a_{A} ,$$

To adjust for various circumstances under which personal moods may fluctuate, we included factors at ego, alter/tie and contact levels as covariates in both models. The ego-level covariates are age group, gender, and Big Five personality traits. The alter- or tie-level covariates involve gender homophily (same gender between ego and alter), alter’s status ranking relative to ego (either higher or lower than ego, with the same rank as the reference), whether ego knew alter very well (a strong tie) and the degree of alter’s embeddedness within ego’s network (percentage of alters in ego’s network that a specific alter personally knew). The contact-level attributes comprise the following factors: whether the contact was initiated by ego, by alter, or by chance, with “by appointment” being the reference category; whether the contact took place in person, as compared with all other modes; whether other people were around during the contact; whether the contact lasted longer than an hour; whether the contact took place in the afternoon or evening(night), compared to the morning; whether the content of the contact was mainly about work, leisure, social chat, or daily routine, compared with other types of contact. For each contact we also controlled for the extent to which the ego reported perceiving that the contact led to an instrumental gain or loss. Response choices ranged from “a great deal” “somewhat”, to “loss”, with “hardly any” as the reference.

Finally, we pooled the results of models with the 100 imputed datasets (Tables 2 and 3). For the *b*th imputed dataset of self-reported mood states, we denoted the estimate and standard error of the *l*th model parameter by ${\widehat{\theta }}_{l}^{(b)}$ and ${\widehat{s}}_{l}^{(b)}$, respectively, for $b=\mathrm{1,2},\dots ,100$. We reported the point estimate of the parameter ${\theta }_{l}$ by the average of these 100 ${\widehat{\theta }}_{l}^{(b)}$ with the standard error equal to the square root of the sample mean of these 100 ${\widehat{s}}_{l}^{(b)}$ squared plus the sample variance of these 100 ${\widehat{\theta }}_{l}^{(b)}$.

In sum, we have followed the reporting guidelines of both contact diary research and ambulatory assessment studies to explain the methods and procedures we used in the current study^24,50^. These procedures cover issues regarding sample selection and size, sampling design, selection and reporting of measures, devices and software used, compliance, data management and analysis, participant training, monitoring, and remuneration.

### Participant involvement

The participants were involved in neither the development of the research questions nor the design of the study. None of the participants was involved in conducting the study nor was asked to provide input in the writing of this manuscript. There are no plans to disseminate the results of the research to the participants.

## Supplementary Information

Supplementary Information 1.

Supplementary Information 2.

## Data Availability

The datasets and the codes used in this study were deposited in a publicly available website, figshare.com. There were four separate links for one figure and three tables listed. Digital Object Identifier (DOI) of Fig. 1: 100.6084/m9.figshare.14183084; DOI of Table 1: 100.6084/m9.figshare.14183099; DOI of Table 2: 100.6084/m9.figshare.14183102; DOI of Table 3: 100.6084/m9.figshare.14183111.
