# Supplementary material for: Using contact tracing from interlocking diaries to map mood contagion along network chains
Source: Sci Rep. 2022 Mar 1;12:3400. doi: 10.1038/s41598-022-07402-1 (PMC8888769; doi:10.1038/s41598-022-07402-1)
Supplement: Supplementary file 1 — Supplementary Information 1. [file 41598_2022_7402_MOESM1_ESM.docx]

Supplement A.

Content of ClickDiary – from the platform of the contact diary used in the study.

1. Questions and response categories about each contact person (all diary keepers answered each of these one-off questions for each contact person they recorded):
2. What is the person’s relationship with you? (1) family (2) relative (3) class mates (4) co-workers (5) close friend (6) just friend (7) indirect tie (8) others.
3. Is the contact person’s relative status higher or lower than you? (1) higher (2) about the same (3) lower (4) not applicable (e.g., neighbors, indirect ties, etc.).
4. What is the person’s gender? (1) male (2) female (3) others (4) I do not know.
5. What is the person’s age (approximately)? (0) 9 or younger 10 (1) 10-19 (2) 20-29 (3) 30-39 (4) 40-49 (5) 50-59 (6) 60-69 (7) 70-79 (8) 80-89 (9) 90 or older (10) I do not know.
6. What is the person’s current work status? (1) jobless (2) working (3) student (4) homemaker (5) retired (6) I do not know.
7. What is the person’s education level? (1) elementary school or below (2) middle school (3) high school (4) junior college (5) university (6) graduate school (9) I do not know.
8. What is the person’s marital status? (1) never married (2) married (3) cohabit with partner (4) divorced (5) separated (6) widowed (7) others (8) I do not know.
9. Where does the person live? (Please select city or town’s name from the list, or choose “I do not know”).
10. Questions about the strength of tie between each pair of the contact persons in each participant’s diary (After completing the questions in Part A regarding each new contact person, the diary keeper answered how well this new person knew each of randomly-selected other contact persons already recorded in the diary. The platform prompted with a screen of five previously recorded names for the diary keeper to answer each of the five ties, then gave another five names on the next screen, and the process continues until the diary keeper skipped for the next question or when all the recorded names were used.)
11. How well does this person know each of the following persons? (1) they know each other personally very well (2) they know each other personally, but not very well (3) they do not know each other personally (4) I do not know.
12. Questions and response categories about each contact (“contact person” or “person” refers to the one with whom the diary keeper interacts):

1.     Which of the following parts of the day did the contact take place? (1) morning (2) afternoon (3) evening or night.

2.     How well did you and the person know each other before the contact? (1) we knew each other extremely well (2) we knew each other very well (3) we knew each other somewhat (4) we did not know each other well (5) we did not know each other at all.

3. How often did you meet or communicate (by any means) with the person before this contact? (1) almost everyday (2) at least once a week (3) at least once a month (4) about once a month or several months (5) about once a year or less often (6) never before.

4.     Who initiated the contact? (1) by prior appointment (2) you initiated (3) the contact person initiated (4) by chance or casual encounter.

5.     What was the main mode of this contact? (1) in-person (2) video chat (3) voice only (4) text only.

6.     How many other persons were also present during the contact? (1) 0 (2) 1-2 (3) 3-5 (4) 6 or more persons.

7.     How long did the contact last? (1) less than 1 minute (2) 1-4 minutes (3) 5-14 minutes (4) 15-59 minutes (5) 1-4 hours (6) more than 4 hours.

8.     What was the main purpose of the contact?  (1) work (2) leisure activities (3) socializing and chatting (4) daily routine (5) others.

9.     How much did you benefit from the contact? (Specific gains not related to mood/emotion) (1) a great deal (2) somewhat (3) hardly any (4) a loss.

10.     How were you feeling before the contact? (1) very good (2) good (3) fair (4) bad (5) very bad.

11.  How were you feeling after the contact? (1) very good (2) good (3) fair (4) bad (5) very bad.

12.  How was the contact person feeling before the contact? (1) very good (2) good (3) fair (4) bad (5) very bad (6) do not know.

12.  How was the contact person feeling after the contact? (1) very good (2) good (3) fair (4) bad (5) very bad (6) do not know.

13.  Did the person show any sign of illness during the contact? (1) no sign at all (2) some signs (3) definite signs (4) do not know.
