# Supplementary figures and images for: Using contact tracing from interlocking diaries to map mood contagion along network chains
Source: Sci Rep. 2022 Mar 1;12:3400. doi: 10.1038/s41598-022-07402-1 (PMC8888769; doi:10.1038/s41598-022-07402-1)

Supplement B. The flow chart of screening participants for final analysis


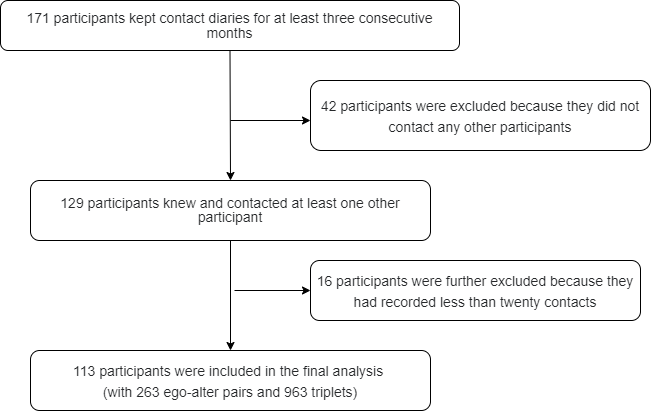

Supplement: Supplementary file 2 — Supplementary Information 2. [file 41598_2022_7402_MOESM2_ESM.docx]
